# Extreme-Aware Time-Series Forecasting via Weak-Label-Guided Mixture of Experts

**DOI:** 10.3390/s26051571

**Published:** 2026-03-02

**Authors:** Jialou Wang, Jacob Sanderson, Wai Lok Woo

**Affiliations:** School of Computer Science, Northumbria University, Newcastle upon Tyne NE1 8ST, UK; jialou2.wang@northumbria.ac.uk (J.W.); jacob.sanderson@northumbria.ac.uk (J.S.)

**Keywords:** extreme-aware time-series forecasting, heuristic unsupervised routing clustering, weak-label-guided mixture of experts

## Abstract

Deep time-series forecasting models can achieve strong average accuracy under normal conditions, yet they often struggle with rare, high-impact extremes, where severe class imbalance biases learning toward majority dynamics. Although infrequent, these extremes frequently correspond to critical events such as natural disasters or power outages. We address this challenge with a weak-label-guided mixture of experts (WL-MoE) that routes each input window to lightweight specialists designed to capture distinct temporal regimes. To prevent routing collapse during early optimisation, WL-MoE follows a two-stage training curriculum. In Stage I, cluster-derived weak labels encourage diverse expert utilisation and promote specialisation under imbalance. In Stage II, guidance is removed and training proceeds solely with the forecasting objective, ensuring that inferences remain fully data-driven. The expert-based structure also supports interpretable routing via expert-usage profiling, enabling regime-level auditing of model behaviour in high-stakes settings. Across seven benchmark datasets, WL-MoE reduces the average MSE by approximately 7.9% and the extreme-case MSE by approximately 23.58% relative to the best baseline. In a UK flood forecasting study, it reduces the all-water MSE by 31.6% and the high-water MSE by approximately 35.0%. These results indicate that weak-label guidance can stabilise specialisation and improve reliability under rare extremes while keeping model behaviour auditable for real-world deployment.

## 1. Introduction

Time-series forecasting supports a wide range of scientific and operational decisions. The problem is that models often break down on rare, high-impact outliers that matter most [1]. In many applications, this is simply a severe imbalance setting [2]. Most observations come from stable dynamics, while extremes appear only occasionally. If we train by minimising the average loss, the majority regime dominates the gradients. The model then learns to do well on typical conditions and can still behave poorly when extreme events occur [3].

These extremes are not just difficult samples. They often coincide with critical situations such as natural disasters or major service disruptions, where forecasts are used to support critical decisions [1,4]. In this context, good average accuracy is not enough. A model can look strong on aggregate metrics and still fail in the tail. That failure is exactly what practitioners care about [3].

A second issue is transparency. When actions depend on forecasts under uncertainty, stakeholders need to judge whether a prediction is reliable and what evidence supports it. This is especially important for extreme predictions, where decisions may involve issuing warnings or allocating resources. If the model is a black box, it becomes hard to trust the output, hard to diagnose mistakes, and hard to justify responses [5,6].

**Figure 1:**
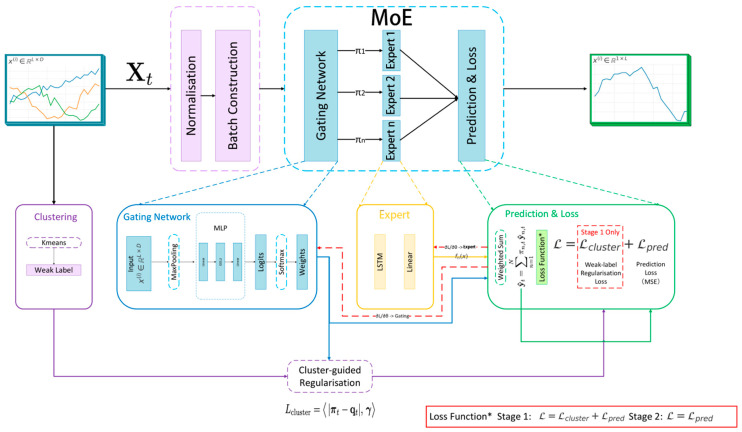
Overview of the proposed weak-label-guided mixture-of-experts (WL-MoE) framework. Each input time series is first normalised and segmented into fixed-length batches. The gating network processes the sequence through max pooling followed by a two-layer MLP, producing logits that are converted into routing weights via softmax. During Stage 1 training, these weights are additionally constrained by cluster-guided regularisation, where unsupervised K-means cluster assignments provide weak labels to stabilise the gating and prevent collapse; in Stage 2 training, the model relies solely on prediction loss. The experts are lightweight LSTM-based predictors, each mapping the input sequence to a forecast horizon. The fusion and loss module computes a weighted sum of expert outputs using the gating weights to form the final prediction $\hat{y}$. Prediction loss (MSE) is computed against ground truth targets, while in Stage 1, it is combined with cluster-guided regularisation. Gradients are backpropagated both to the experts and to the gating network, enabling end-to-end optimisation. Colors indicate functional sections: purple denotes preprocessing and weak-label components, blue denotes gating/routing components, yellow denotes expert components, and green denotes prediction-and-loss components.

To address these challenges, we propose the weak-label-guided mixture of experts (WL-MoE, Figure 1). The a priori motivation for using an MoE architecture is that extreme-aware forecasting is intrinsically heterogeneous: Normal and extreme regimes follow different temporal dynamics and are difficult to model well with a single shared predictor. MoE offers conditional computation through specialised experts with learned routing, enabling regime-wise capacity allocation while retaining end-to-end training [7,8,9,10]. WL-MoE itself is implemented as a deep-learning model with neural experts and a neural gate; the contribution is not to remove modelling assumptions but to improve specialist allocation and training stability under severe imbalance.

However, severe imbalance makes standard mixture models unstable. Training tends to favour the expert that best fits the majority regime. Routing concentrates on that expert, while experts that should model rare extremes receive too few updates and remain undertrained. This is the gate collapse problem [11,12,13].

Accordingly, the research aim of this study is to evaluate whether weak-label-guided routing can stabilise MoE specialisation under severe imbalance and improve both overall forecasting accuracy and tail-event reliability without sacrificing interpretability.

Relative to current SOTA forecasters, our method is positioned as a training and routing framework that is complementary to strong sequence backbones rather than a replacement for them. Existing SOTA models, including Transformer-based and MLP-based forecasters, achieve strong average-case accuracy but can still suffer on rare tail regimes under severe imbalance [14,15,16,17,18,19]. In contrast, WL-MoE focuses on regime-wise capacity allocation and stable expert specialisation for minority dynamics, beyond output-level reweighting or resampling alone [4,20,21]. The core motivation is that MoE is naturally suited to datasets with highly heterogeneous temporal behaviours, where different experts can specialise in distinct regimes within the same dataset through conditional routing [7,8,9,10]. Under severe imbalance, this potential can be undermined by gate collapse, so we use cluster-derived weak labels as a practical training scaffold to stabilise early specialisation and then remove guidance during fine-tuning [22,23,24].

WL-MoE reduces collapse through a two-stage strategy. In the first stage, we guide routing using weak labels derived from unsupervised clustering. These labels are not treated as ground truth. They only provide a coarse cue about temporal patterns, and they encourage the gate to spread samples across experts early in training. This gives each expert enough signal to start specialising. In the second stage, we remove weak-label guidance and continue training only on the forecasting objective. The final model is therefore fully data-driven at inference, but it benefits from more stable specialisation during optimisation.

WL-MoE also supports interpretability through its expert-based structure. Because the gating network routes each input to specific experts, we can profile expert usage over time and across covariates to reveal how different temporal behaviours are handled. This expert profiling offers a practical way to audit model behaviour, understand specialisation, and diagnose potential failure modes during extreme events.

In summary, the contributions of this paper are three-fold:

1. We propose WL-MoE, a mixture-of-experts forecasting architecture that decomposes time-series modelling into specialist experts, enabling dedicated modelling of different temporal dynamics.
2. We introduce a stabilised two-stage training procedure that prevents gate collapse under severe imbalance using cluster-derived weak labels to encourage early expert specialisation, followed by purely data-driven fine-tuning.
3. We demonstrate that WL-MoE supports interpretability via expert-usage profiling, providing insight into how experts specialise across temporal regimes and how routing changes during extreme events.

The remainder of this paper is organised as follows. Section 2 reviews related work on imbalanced forecasting, tail-aware objectives, mixture routing stability, and time-series interpretability. Section 3 presents WL-MoE, including weak-label generation and the two-stage training procedure. Section 4 reports experimental results and diagnostic analyses, and Section 5 presents an empirical flood forecasting study. Section 6 and Section 7 discuss limitations and practical considerations, followed by conclusions in Section 8.

## 2. Related Work

Research on time-series forecasting spans a long trajectory from classical statistical approaches to modern deep learning architectures. Early methods such as ARIMA [25] and state-space models, including the Kalman filter (KF) [26], hidden Markov models (HMMs) [27], and dynamic linear models (DLMs) [28], remain attractive due to their interpretability and computational efficiency. In practice, exponential smoothing and related methods are also widely used as strong statistical baselines [29], and large-scale production systems often rely on models designed for stable deployment and human-facing diagnostics [5]. However, these approaches are typically constrained by linearity, stationarity, or carefully specified noise assumptions [30], which limits their ability to represent non-linear dynamics, abrupt shifts, and rare extremes. Deep learning has become dominant for forecasting, with recurrent, convolutional, and attention-based architectures driving recent progress. Recurrent models such as long short-term memory(LSTM) provide a flexible way to model temporal dependencies, and probabilistic recurrent neural network (RNN) forecasters have been widely adopted for uncertainty-aware prediction [31]. Convolutional approaches, including temporal convolutional networks, offer efficient receptive field growth and strong performance on many sequence modelling tasks [32]. More recently, attention-based models such as Informer, Autoformer, and FEDformer have set strong baselines on long-horizon forecasting benchmarks [14,15,16]. In parallel, simple multi-layer perceptron (MLP)-style designs challenge the necessity of attention. TSMixer and PatchMixer match or surpass Transformer variants on standard datasets while improving efficiency [17,18]. Other deep forecasting lines further broaden the design space, including residual and basis-expansion models [33] and architectures that combine attention with structured temporal feature selection for multi-horizon prediction [6,34], enhancing interpretability. Beyond intrinsic architectural transparency, post hoc explanation methods aim to clarify model behaviour. In tabular—and increasingly in spatial—domains, attribution-based methods such as Grad-CAM highlight influential spatial regions in deep models and have been applied in environmental risk modelling to support the qualitative validation of predictions [2,35,36]. Counterfactual explanations are also widely used, which identify minimal changes to an input that would alter a model’s prediction, thereby exposing decision boundaries and model sensitivities [37,38]. While these approaches can offer insights into model predictions, they are less naturally aligned with structured sequence forecasting tasks. In contrast, our approach leverages the internal routing structure of a mixture-of-experts model to provide interpretability through expert-usage profiling.

Despite this progress in temporal forecast models, most architectures are still optimised for average-case error. Under severe imbalance, this frequently results in the systematic under-prediction of rare extremes [19]. A growing body of literature treats this as an imbalanced regression problem, where target values near the tail are sparsely represented and thus receive limited learning signal. Typical strategies include reweighting, resampling, and synthetic oversampling tailored to continuous targets [20,21]. Recent work also considers tail-event analysis as a first-class objective, proposing specialised training and evaluation protocols for rare events in time series [4,39]. Distributional and quantile forecasting further supports risk-sensitive decision-making by predicting uncertainty or conditional quantiles instead of point estimates [40]. However, these approaches can still be sensitive to dataset heterogeneity, and it remains challenging to ensure that a model develops distinct internal competencies for minority regimes rather than merely shifting errors. A related line of work addresses heterogeneity through divide-and-conquer modelling. Mixture-of-experts (MoE) frameworks implement conditional computation by routing each input to one or more specialised submodels, enabling different components to focus on different patterns [7,8]. Classical MoE formulations commonly use soft gating over all experts, while many recent large-scale sparse MoE systems adopt top-*k* activation to reduce computation. Modern sparse MoE designs scale this idea via learned routing with a small active subset of experts per input, allowing large model capacity without proportional compute [9]. Recent systems demonstrate strong scaling in language models [10] and analogous benefits in vision and reinforcement learning through regime- or task-specific specialisation [41]. Scholars similarly treat prediction as a divide-and-conquer process via a learned gate that routes inputs to specialised submodels [7]. These results suggest that routing-based architectures are a promising mechanism for handling heterogeneous temporal behaviours, including conditions associated with extremes. However, training-routed ensembles often suffer from gate collapse, where the router concentrates most inputs on a small subset of experts, leaving others under-utilised or effectively inactive. This issue is widely reported in practical MoE training, and analyses show that collapse can manifest as routing imbalance, dead experts, and reduced effective capacity [11,12,13]. Standard remedies include auxiliary balancing losses, regularisation, and softmax routing, but imbalance in the underlying data distribution can still exacerbate minority-expert collapse by depriving experts of sufficient gradient signal [10]. This is particularly problematic for extreme-aware forecasting, where the goal is precisely to allocate capacity to rare temporal behaviours. To stabilise specialisation without requiring manual regime annotation, weak supervision and pseudo-labelling provide a natural bridge. Pseudo-labelling and related weak-label strategies have a long history as a way to inject coarse structure early in training while keeping the final objective fully data-driven [22]. In the time-series setting, unsupervised clustering offers a practical mechanism for producing coarse pattern indicators, and a substantial body of literature studies clustering objectives and shape-based similarity for temporal sequences [23,24]. The proposed training strategy follows two stages: In Stage I, cluster-derived weak labels provide coarse routing cues to encourage broader expert utilisation and reduce early gate collapse; in Stage II, weak-label guidance is removed, and optimisation proceeds only with the forecasting objective, so inference remains fully data-driven. In summary, all forecasting families rely on modelling assumptions, but their limitations under severe imbalance differ by approach. Classical models are often constrained by explicit structural assumptions; deep models and routed ensembles can still be dominated by majority-regime optimisation and gate imbalance; rebalancing strategies may lack robustness across heterogeneous datasets. Our approach therefore builds on deep MoE modelling assumptions while targeting a specific optimisation problem: stable minority-regime specialisation under rare extremes.

## 3. Materials and Methods

### 3.1. Problem Formulation

Let $D={\left\{d_{i}\in R^{C}\right\}}_{i=1}^{I}$ denote a multivariate time series of length *I* with *C* covariates. Given a historical window of length *L*, the task is to predict the subsequent *H*-step future trajectory, formally expressed as Equation (1):

(1) $${\hat{y}}_{i+1:i+H}=f_{\Theta }\;\left(D_{i-L+1:i}\right),$$

where $f_{\Theta }\left(\cdot \right)$ is a forecasting model parameterised by Θ, and $y_{i+1:i+H}\in R^{H}$ denotes the ground-truth targets.

The training dataset is constructed from sliding windows, yielding paired instances of input and target sequences:

(2) $$X={\left\{\left(D_{i-L+1:i},\;y_{i+1:i+H}\right)\right\}}_{i=L}^{I-H}$$

where *X* denotes the training set constructed with length-*L* sliding windows, and T=I−L−H+1 is the total number of samples. The *i*-th sample consists of the input segment $D_{i-L+1:i}$ and the target segment $y_{i+1:i+H}$. Equation (2) ensures that each training pair is aligned in time, preserving the temporal ordering required for sequence prediction.

To train the model, we minimise the mean squared error (MSE) between predicted and observed sequences that penalises large deviations at each horizon. This objective serves as the baseline loss onto which our weak-label-guided mixture-of-experts framework adds additional regularisation terms.

### 3.2. Mixture-of-Experts Framework

Instead of relying on a single predictor, we employ a mixture-of-experts (MoE) architecture. Suppose that we instantiate *N* specialised forecasting experts ${\left\{f_{n}\right\}}_{n=1}^{N}$, each producing an individual forecast at every time step *t*, given the same input history $X_{t}$:

(3) $${\hat{y}}_{t}^{n}=f_{n}\left(X_{t}\right)\;$$

Equation (3) allows each expert to capture distinct temporal regimes.

In this work, each expert uses a classical LSTM predictor. This study’s focus is the effect of MoE routing and weak-label guidance under severe imbalance, so the expert backbone is intentionally fixed rather than tuned across recurrent variants. GRU-based experts and other sequence backbones are valid alternatives, but they are outside the main scope of this paper. To combine these outputs, we introduce a gating network g(·), which maps the shared input history $X_{t}$ to a vector of unnormalised routing scores $z_{t}\in R^{N}$ over the *N* experts:

(4) $$z_{t}=g\left(X_{t}\right),$$

where $z_{t}=\left(z_{1,t},\ldots ,z_{N,t}\right)$ denotes the routing logits at time step *t*. The gating network is implemented as a lightweight neural router. Given the input window $X_{t}\in R^{L\times C}$, we first apply temporal max pooling over the length dimension to obtain a fixed-size feature vector $v_{t}\in R^{C}$. The vector $v_{t}$ is then passed through a two-layer MLP to produce the routing logits $z_{t}$. Routing is dense (not top-*k* sparse), so all experts participate through the softmax weights in Equation (5). For completeness, sparse top-*k* routing retains only the *k* largest routing logits and renormalises the corresponding probabilities. Let $S_{t}$ be the selected top-*k* expert set at sample *t*; then, one can define ${\tilde{\pi }}_{n,t}=\frac{\pi_{n,t}\;1\left[n\in S_{t}\right]}{\sum_{j\in S_{t}}\pi_{j,t}}$. In this work, we use dense routing (k=N) because the expert pool is moderate and the objective emphasises stable minority-regime learning under severe imbalance, where hard truncation can suppress gradient flow to under-selected experts in early training. These logits are subsequently converted into mixture weights through the softmax function:

(5) $$\pi_{n,t}=\frac{\exp(z_{n,t})}{\sum_{j=1}^{N}\exp\left(z_{j,t}\right)},\;n=1,\ldots ,N,\;\pi_{t}\in \Delta^{N-1},$$

where the denominator sums over all experts indexed by *j*, $\pi_{t}=\left(\pi_{1,t},\ldots ,\pi_{N,t}\right)$, and $\Delta^{N-1}$ denotes the (N−1)-dimensional probability simplex. Equation (5) ensures that each component $\pi_{n,t}$ is non-negative and that the weights sum to one.

The final prediction is obtained as the convex combination of expert outputs:

(6) $${\hat{y}}_{t}=\sum_{n=1}^{N}\pi_{n,t}\;{\hat{y}}_{n,t}$$

Equation (6) integrates the specialised knowledge of individual experts, while the gating network in (4) and (5) adapts based on the input regime. This differs from a static meta-ensemble: Routing weights are input-dependent for each sample and are learned jointly with expert parameters through end-to-end optimisation. Component-wise exclusion should therefore be interpreted by role. Removing the gating module does not yield an ablated MoE variant; it changes the model class to a static ensemble without input-dependent routing. By contrast, removing weak-label guidance is feasible and corresponds to unguided MoE training, which, in severe imbalance settings, is more prone to routing concentration and expert under-utilisation during early optimisation.

### 3.3. Weak-Label Generation

Weak labels are introduced only for Stage I training to stabilise early expert specialisation under severe imbalance. They are obtained from the same input segments $X_{t}$ used by the model.

We use K-means as the weak-label generator because Stage I requires a simple, deterministic, and scalable partition of input windows rather than another high-capacity model. K-means provides centroid-based assignments with low computational overhead, straightforward implementation across large benchmarks, and stable one-hot labels that map directly to the cluster-guidance term in Equation (10). This keeps weak supervision lightweight and reproducible while avoiding additional modelling complexity that could confound routing analysis. The weak labels are used only as temporary optimisation scaffolding in Stage I and are removed in Stage II, so the final inference does not depend on K-means assumptions.

In this work, the feature mapping ϕ(·) used for clustering is defined as a direct vectorisation of each standardised input window. For $X_{t}\in R^{L\times C}$, we use

(7) $$\phi \left(X_{t}\right)=\mathrm{vec}\left(X_{t}\right)\in R^{LC},\;$$

where vec(·) stacks the time-major entries of $X_{t}$ into a single feature vector. No handcrafted summary statistics are used; clustering is performed on the full standardised window representation. This preserves complete temporal-covariate information for K-means-based weak-label construction and makes the procedure fully reproducible. Practically, this vectorisation has linear cost in both memory and runtime with dataset size and window dimensionality (storing roughly T×LC values and repeatedly computing distances in $R^{LC}$ during K-means iterations). To keep preprocessing scalable on larger datasets, we build vectors in batches and fit K-means with mini-batch/chunked updates, which reduces peak memory and wall-clock time without changing the weak-label definition.

We generate weak labels by clustering input segments in a feature space induced by a fixed mapping ϕ(·). Concretely, for each training sample, we compute a feature representation $\phi (X_{t})$, and apply *K*-means clustering to partition these representations into *K* coarse temporal patterns. The resulting cluster assignment $c_{t}$ is then encoded as a one-hot vector $q_{t}\in {\left\{0,1\right\}}^{K}$, which serves as the weak label used for guidance in Stage I. In our implementation, the number of clusters is set to match the number of experts (K=N), so cluster-derived weak labels align directly with expert-routing targets. For each sensitivity setting N∈{3,9,18}, we set K=N. Stage-I weak labels are constructed with a fixed pipeline: Training windows are formed from the Stage-I training split, each variate is standardised using training-split statistics, and each window is mapped by time-major vectorisation $\phi \left(X_{t}\right)=\mathrm{vec}\left(X_{t}\right)$. K-means is then fit once on these vectors with K=N using the Euclidean objective in Equation (8). Each sample is assigned to its nearest centroid and encoded as a hard one-hot weak label per window (not soft/distributed labels), which remains fixed throughout Stage-I training. The complete Stage-I weak-label generation procedure is summarised in Algorithm 1.

### 3.4. Weak-Label Guidance

Weak labels $q_{t}$ are generated from historical windows, as described in Section 3.3. Direct optimisation of the prediction loss in Equations (6) and (19) often causes the gating network to collapse, i.e., most samples are routed to a single expert while others remain inactive. To mitigate this, we introduce weak labels derived from unsupervised clustering as auxiliary guidance.
**Algorithm 1:** Weak-Label Generation (Stage I Only)**Require:** Training samples X from Equation (2), number of clusters *K*, feature mapping ϕ(·)**Ensure:** Weak labels ${\left\{q_{t}\right\}}_{t=1}^{T}$ and assignments ${\left\{c_{t}\right\}}_{t=1}^{T}$1:Compute feature vectors $\phi (X_{t})$ for each training input segment $X_{t}$2:Fit *K*-means on ${\left\{\phi \left(X_{t}\right)\right\}}_{t=1}^{T}$ to obtain centroids ${\left\{\mu_{k}\right\}}_{k=1}^{K}$3:**for **t=1 to *T* **do**4:    $c_{t}\leftarrow \arg\min_{k}{∥\phi \left(X_{t}\right)-\mu_{k}∥}_{2}^{2}$5:    Set $q_{t}$ as the one-hot encoding of $c_{t}$6:**end for**

Given an input segment $X_{t}$, we compute $v_{t}=\phi \left(X_{t}\right)$ and apply *K*-means in the feature space to obtain a coarse regime assignment:

(8) $$c_{t}=\arg\min_{k\in \{1,\ldots ,K\}}\;{∥v_{t}-\mu_{k}∥}_{2}^{2}$$

where $\mu_{k}$ is the centroid of cluster *k*. The assignment $c_{t}$ is encoded as a hard one-hot vector $q_{t}\in {\left\{0,1\right\}}^{K}$ for sample *t* (not soft/distributed labels). Equation (8) provides only heuristic guidance and does not constitute ground-truth annotation.

To integrate weak labels with gating, we define inverse-frequency weights:

(9) $$\rho_{k}=\frac{\left|\{\;t:c_{t}=k\;\}\right|}{T},\;\gamma_{k}=\frac{1}{\rho_{k}+\varepsilon_{\mathrm{reg}}},\;$$

where $\rho_{k}$ is the frequency of cluster *k* in the Stage-I training set, $\varepsilon_{\mathrm{reg}}>0$ is a smoothing constant to avoid division by zero, and $\gamma_{k}$ up-weights under-represented clusters.

The sample-level cluster regularisation term is

(10) $$L_{\mathrm{cluster},t}=\sum_{k=1}^{K}\gamma_{k}\;|\pi_{k,t}-q_{k,t}|,\;$$

which encourages the gating distribution $\pi_{t}$ from Equation (5) to align with the weak label $q_{t}$ while compensating for imbalance.

To understand why this regularisation prevents gate collapse, we analyse the optimisation dynamics. Using $\rho_{k}$ from Equation (9), without weak-label guidance, the expected gradient magnitude for expert *k* (in one-to-one correspondence with cluster *k*) is proportional to $\rho_{k}$, leading to the following concentration inequality:

(11) $$E_{t}\;\left[\pi_{k,t}\right]\;\geq \;\frac{\rho_{k}}{\sum_{j:\;\rho_{j}\geq \rho_{k}}\rho_{j}}\;\geq \;\rho_{k},\;$$

where $\pi_{k,t}$ is the routing probability assigned to expert *k* at sample *t*, the expectation $E_{t}\left[\cdot \right]$ is taken over training samples *t*, and the denominator sums over all clusters *j* for which their frequencies $\rho_{j}$ are at least $\rho_{k}$. This inequality demonstrates that experts corresponding to rare clusters (small $\rho_{k}$) receive rapidly diminishing routing probabilities as training progresses.

The cluster regularisation modifies this dynamic by introducing a corrective bias. With Stage-I objective $L^{\left(1\right)}$, the gradient with respect to gating logit $z_{k,t}$ can be written as

(12) $$\frac{\partial L^{\left(1\right)}}{\partial z_{k,t}}=\frac{\partial L_{\mathrm{MSE}}}{\partial z_{k,t}}+\lambda_{\mathrm{wl}}\sum_{j=1}^{K}\gamma_{j}\;\mathrm{sign}\left(\pi_{j,t}-q_{j,t}\right)\frac{\partial \pi_{j,t}}{\partial z_{k,t}},$$

where $\lambda_{\mathrm{wl}}>0$ is the weak-label regularisation weight.

The key insight from Equation (12) is that the regularisation term provides a corrective force scaling inversely with cluster frequency, ensuring that rare clusters receive disproportionately strong gradient signals.

From an information-theoretic perspective, we quantify expert utilisation using the entropy of the gating distribution:

(13) $$H\left(\pi_{t}\right)=-\sum_{n=1}^{N}\pi_{n,t}\log\pi_{n,t}\;$$

Gate collapse corresponds to $H(\pi_{t})\to 0$, while uniform utilisation yields $H(\pi_{t})=\log K$. We can establish a lower bound on entropy during Stage 1 training. Let $\delta \;=\;\min_{k}\gamma_{k}^{-1}$ (minimum inverse weight):

(14) $$H\left(\pi_{t}\right)\;\geq \;\log\;\left(N\;\delta +1\right)\;-\;\delta \log\delta ,$$

where the bound follows from the constraint that $\pi_{n,t}\geq \frac{\delta }{N\delta +1}$ for all *n*. Equation (14) thus provides a non-trivial lower bound on the gating entropy, preventing complete collapse ($H(\pi_{t})\;\to \;0$).

By combining the prediction objective described in Equation (19) with the weak-label regulariser in Equation (10), the gating network is prevented from degenerating into trivial routing and each expert receives meaningful gradient signals during training.

### 3.5. Routing

Our mixture uses a gating network to compute routing logits $z_{t}\in R^{N}$ from the input segment $X_{t}$. The logits are converted into mixture weights $\pi_{t}$ via a softmax function, and the final prediction is formed as a weighted combination of expert forecasts. The exact computation of $\pi_{t}$ and ${\hat{y}}_{t}$ is summarised in Algorithm 2.
**Algorithm 2:** Forward Propagation with Softmax Routing**Require:** Input segment $X_{t}$, number of experts *N***Ensure:** Prediction ${\hat{y}}_{t}$ and mixture weights $\pi_{t}$1:$z_{t}\leftarrow g\left(X_{t}\right)$2:$\pi_{t}\leftarrow \mathrm{softmax}\left(z_{t}\right)$3:**for**n=1 to *N* **do**4:    ${\hat{y}}_{n,t}\leftarrow f_{n}\left(X_{t}\right)$5:**end for**6:${\hat{y}}_{t}\leftarrow \sum_{n=1}^{N}\pi_{n,t}{\hat{y}}_{n,t}$7:**return**${\hat{y}}_{t},\pi_{t}$

### 3.6. Two-Stage Training Strategy

To stabilise learning, we adopt a two-stage training procedure. Stage 1 incorporates weak-label guidance from (10), while Stage 2 removes this auxiliary guidance and relies solely on predictive accuracy.

In Stage 1, the objective combines prediction loss with the cluster regulariser (10):

(15) $$L^{\left(1\right)}=L_{\mathrm{MSE}}+\lambda_{\mathrm{wl}}\;\frac{1}{T}\sum_{t=1}^{T}L_{\mathrm{cluster},t}$$

Equation (15) encourages the gating network from (5) to respect the cluster structure during early training, thereby preventing routing collapse. Here, $\lambda_{\mathrm{wl}}$ controls the strength of weak-label guidance relative to prediction error, and $\varepsilon_{\mathrm{reg}}$ in Equation (9) smooths inverse-frequency weights for rare clusters. No additional auxiliary penalties are used in Stage 1 beyond the weighted cluster term.

After Stage 1 has seeded expert diversity, Stage 2 fine-tunes the model by optimising only the predictive term:

(16) $$L^{\left(2\right)}=L_{\mathrm{MSE}}.$$

Equation (16) removes auxiliary constraints, allowing the experts to adapt freely while retaining the structural diversity established in Stage 1.

Together, (15) and (16) define a curriculum: weak-label guidance is used as a scaffold during early training, while the final model is driven exclusively by end-to-end predictive accuracy.

### 3.7. Expert-Oriented Explainability

To build trust in WL-MoE and to verify that the two-stage training yields meaningful task decomposition, we focus on expert-oriented explainability through routing behaviour. Specifically, we profile expert usage over evaluation partitions to quantify specialisation and detect potential routing collapse.

#### Expert-Usage Profiling

Given an evaluation partition ${\left\{D_{k}\right\}}_{k=1}^{K}$ (e.g., time ranges, covariate buckets, or weak-label groups), we compute the expert-usage matrix as in (17).

(17) $$U_{n,k}\;=\;\frac{1}{|D_{k}|}\sum_{t\in D_{k}}\pi_{n,t},\;U\in R^{N\times K}.$$

Concentration in rows or columns of (17) indicates that certain experts are preferentially activated for particular subsets, providing a transparent view of specialisation across conditions.

To quantify routing diversity at the sample level, we use the routing entropy in (13) and optionally its partition average in (18). Lower values of (13) indicate concentrated routing, while higher values indicate more even utilisation.

(18) $$\bar{H}\left(D_{k}\right)=\frac{1}{|D_{k}|}\sum_{t\in D_{k}}H\left(\pi_{t}\right).$$

Entropy routing is used here as a confidence-and-diversity indicator for the gate. When routing mass is concentrated on one expert, entropy approaches zero; when routing is more evenly distributed across experts, entropy increases toward the uniform-routing limit. We, therefore, use sample-level entropy to characterise local routing sharpness and partition-level average entropy to compare routing behaviour across regimes. Interpreted together with the expert-usage matrix, these statistics help distinguish healthy regime-dependent specialisation from collapse-like behaviour.

## 4. Experiment

### 4.1. Datasets

We conduct experiments on seven widely used forecasting benchmarks spanning diverse domains and temporal resolutions—ETTh1/2 (seven variables, L=17,420, hourly), ETTm1/2 (seven variables, L=69,680, 15 min), Weather (21 variables, L=52,696, 10 min), Electricity (321 variables, L=26,304, hourly), and Traffic (862 variables, L=17,544, hourly). Here, *C* denotes the number of variables, and *L* denotes the number of time steps. The benchmark datasets are commonly evaluated under two forecasting protocols. In the multivariate setting, the model predicts multiple target series simultaneously. In the univariate setting, the model focuses on forecasting a single target series at a time. Both settings have been extensively studied in the literature [17,42,43]. In this work, we adopt the univariate formulation, which enables a clearer partition of extreme cases and more transparent evaluation of robustness. Our primary motivation is that extreme events, though rare, are of disproportionate practical importance, and the univariate setting allows us to isolate and assess forecasting performance under such conditions more rigorously.

Event definitions differ between benchmark and application-specific settings in this study. For the seven public benchmarks (ETTh1/2, ETTm1/2, Weather, Electricity, and Traffic), an event is defined statistically as an upper-tail excursion of the selected target variable under the extreme-case partition rule defined below. This captures rare distributional regimes without imposing a specific physical incident label, so benchmark results are interpreted as statistical tail-robustness evaluation. In the flood forecasting study, events have direct physical meaning and correspond to high-water episodes in river level forecasting (Section 5).

#### Extreme Case Partitioning

**Figure 2:**
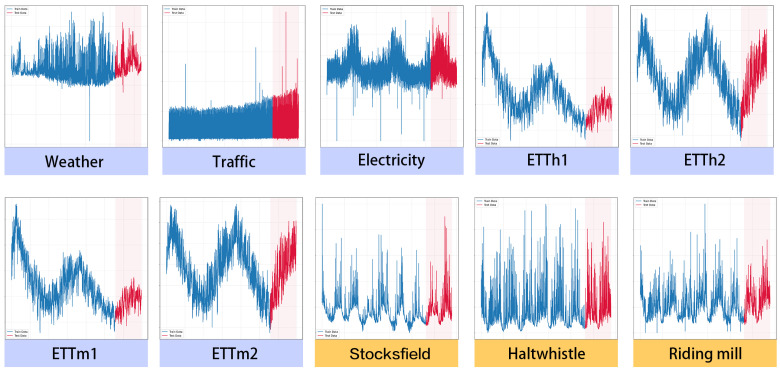
Visual comparison of target series across datasets. Each panel shows the target time series; the blue segment is the training portion, and the red segment is the test portion. Panels with purple labels are the benchmark datasets, whereas panels with yellow labels are the flood-water-level datasets.

To evaluate robustness under rare but impactful conditions, we construct an extreme subset of the test set. Specifically, for a test sequence $y_{t}$, we first compute the 95th percentile of the training distribution of the target series, denoted $\tau_{0.95}$. If more than 5% of the values in $y_{t}$ exceed $\tau_{0.95}$, the sequence is labelled as an extreme case. The rule is applied to each forecast window in a deterministic way. For a horizon of length *H*, define the exceedance count as $m_{t}=\sum_{h=1}^{H}I\left(y_{t+h}>\tau_{0.95}\right)$. A window is assigned to the extreme subset when $m_{t}/H>0.05$. This gives an explicit threshold per horizon (for example, at least five exceedances when H=96, at least 10 when H=192, at least 17 when H=336, and at least 37 when H=720). The threshold $\tau_{0.95}$ is estimated from the training split and then fixed for evaluation, so no test information leaks into partitioning. For public benchmarks, this criterion defines statistical upper-tail windows rather than domain-labelled incidents; corresponding tail-concentrated versus regular patterns are illustrated in Figure 2.

### 4.2. Evaluation Metrics

We adopt two standard regression metrics: mean squared error (MSE) and mean absolute error (MAE). For each horizon *H*, the metrics are computed as

(19) $$\begin{array}{cc} \mathrm{MSE} & =\frac{1}{S_{\mathrm{test}}}\sum_{s=1}^{S_{\mathrm{test}}}{∥y\left(s\right)-\hat{y}\left(s\right)∥}_{2}^{2} \end{array}$$

(20) $$\begin{array}{cc} \mathrm{MAE} & =\frac{1}{S_{\mathrm{test}}}\sum_{s=1}^{S_{\mathrm{test}}}{∥y\left(s\right)-\hat{y}\left(s\right)∥}_{1}\; \end{array}$$

where y(t) and $\hat{y}\left(t\right)$ denote the ground-truth and predicted vectors over the horizon. $s=1,\ldots ,S_{\mathrm{test}}$ indexes the evaluation samples, and $S_{\mathrm{test}}$ is the total number of evaluation windows.

Evaluation is conducted by computing the MSE and MAE according to (19) and (20) on both the full test set and the extreme subset. This dual evaluation allows us to assess both overall accuracy and robustness under rare, high-impact conditions.

### 4.3. Implementation Details

We evaluate our approach against five widely used forecasting models: Informer, Autoformer, FEDformer, TimesNet, and TsMixer. These baselines are chosen to cover major design families in multivariate forecasting under one protocol, including sparse-attention Transformer (Informer), decomposition-based Transformer (Autoformer), frequency-enhanced Transformer (FEDformer), convolutional temporal modelling (TimesNet), and MLP-mixing forecasting (TsMixer). This comparison is intended to be representative rather than an exhaustive leaderboard of every newly released model.

For all models, we adopt the same input length L=336 and forecast horizons H∈{96,192,336,720}, following prior work [15]. Each dataset is standardised independently per variate using statistics from the training set. Training, validation, and test splits are consistent with public benchmark protocols.

Optimisation is performed using the Adam. Unless otherwise stated, we set the initial learning rate as $\eta =3\times 10^{-4}$, $\beta_{1}=0.9$, $\beta_{2}=0.999$, and $\epsilon_{\mathrm{adam}}=10^{-8}$. The models are trained in two stages. Each stage uses early stopping (patience = 5) on its own validation objective: Stage 1 monitors the validation value of $L^{\left(1\right)}$ in (15), while Stage 2 monitors the validation MSE in (16). All experiments were implemented in Python (v3.14.0) using PyTorch (v2.9.1), NumPy (v2.3.4), and scikit-learn (v1.7.2).

For comparability, all competing models are run with the same data splits, input/horizon settings, optimizer type, early-stopping rule, and maximum epoch budget in our environment, without model-specific unequal tuning budgets. The gating network’s implementation in experiments follows the design described in Section 3.2. It applies temporal max pooling over each input window, followed by a two-layer MLP that outputs *N* routing logits. The router uses standard softmax normalisation (dense routing without top-*k* truncation), and its parameters are trained jointly with expert parameters in both training stages. To ensure fair comparisons across expert-count settings, we keep the remaining training hyperparameters and backbone settings consistent rather than retuning each configuration separately. Therefore, the selected setting is a reproducible comparative choice under a fixed protocol and not a claim of globally optimal hyperparameter selection.

### 4.4. Results and Discussion

Table 1 reports MSE and MAE for six models on each dataset and horizon. Because these benchmarks differ substantially in scale, variable composition, and noise characteristics, we do not use the arithmetic averages of raw MSE/MAE across datasets for inferential comparison. Instead, we evaluate performance per dataset and per horizon under a common protocol. Our method (WL-MoE) ranks first or second on the vast majority of settings, with more pronounced gains on the extreme-case subset.

Although WL-MoE is consistently first or second on the standard benchmarks, its margin over strong baselines is not dramatic. Figure 2 contrasts the target series across all datasets: The seven benchmarks (image with purple labels) are largely quasi-stationary with smooth seasonal trends and comparatively narrow, slowly varying amplitudes; their held-out segments (red) follow the same regime as the training portion (blue) with only mild right tails. By contrast, the three flood-water-level cases (image with yellow labels) exhibit bursty, heavy-tailed spikes tightly clustered in the test interval, pronounced regime shifts, and long-range dependence.

WL-MoE is most effective on high-detachment datasets—those in which the evaluation mass concentrates in a right-tail regime that is far from the typical behaviour. To quantify this property, we introduce a dataset-level tail detachment score (TDS). Given the target series $\left\{y_{t}\right\}$, we first apply robust normalisation using Equation (21):

(21) $$u_{t}=\frac{y_{t}-\mathrm{median}\left(Y\right)}{\mathrm{IQR}(Y)}\;$$

This normalisation uses the IQR as a robust measure of spread, which is the range covered by the middle 50% of values. The high-tail subset is defined as $U_{\tau }\;=\;\left\{\;u_{t}\;:\;u_{t}\geq Q_{\tau }\left(u\right)\;\right\}$ at threshold τ. The score is described via Equation (22):

(22) $${\mathrm{TDS}}_{\tau }\;=\;\mathrm{median}\left(U_{\tau }\right)-\mathrm{median}\left(u\right)\;$$

where ${\mathrm{TDS}}_{\tau }$ is the tail detachment score at tail threshold τ, $\mathrm{median}(U_{\tau })$ is the median of the extreme cases subset, and median(*u*) denotes the median of the full dataset.

The tail detachment at ${\mathrm{TDS}}_{0.95}$ is substantially higher in floods than benchmarks: floods—Stocksfield 2.80, Haltwhistle 2.60, and Riding Mill 1.53 (mean 2.31); benchmarks—Weather 1.48, Traffic 0.59, Electricity 1.14, ETTh1 1.18, ETTh2 0.83, ETTm1 1.18, and ETTm2 0.83 (mean 1.03). We therefore proceed to the real-data flood study.

## 5. Empirical Study on Flood Prediction

**Figure 3:**
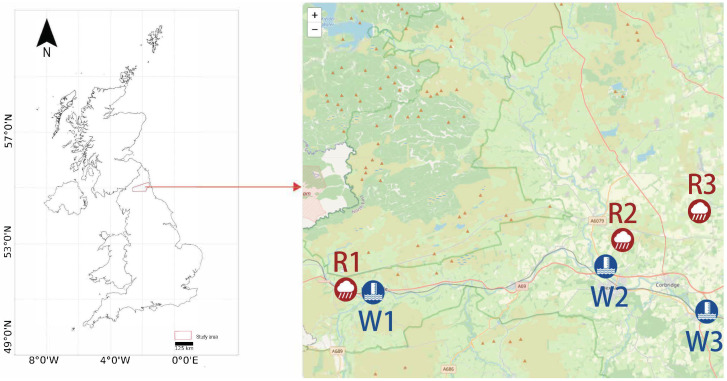
Study area in Northumberland, UK. The **left panel** locates the study region within the UK. The **right panel** shows the local sensor layout. Blue markers denote river stage sites: W1 (Haltwhistle), W2 (Riding Mill), and W3 (Stocksfield). Red markers denote rainfall gauges (R1–R3) used to provide precipitation forcing.

To demonstrate the method in a high-impact operational setting, we evaluate WL-MoE on multi-horizon flood water-level forecasting in Northumberland, UK. We consider three river-level sites, denoted as W1–W3, corresponding to Haltwhistle (W1), Riding Mill (W2), and Stocksfield (W3). Each site is paired with a nearby rainfall gauge (R1–R3) that provides the precipitation forcing. Figure 3 summarises the study area and sensor layout.

Rainfall (mm/15 min) and water level (m/15 min) are obtained from the DEFRA hydrometric archive (https://environment.data.gov.uk/hydrology/explore (accessed on 1 October 2025)) at 15 min resolution. We adopt a strict chronological split, using records up to March 2024 for training and April 2024 to February 2025 for testing. Supervised samples are generated via a sliding window: Each input consists of 32 past rainfall and water-level observations plus 32 future rainfall steps, and the target is the 32-step future water level. The station-specific training start times are determined by data availability: Haltwhistle starts at 1 January 2016 00:00:00, Riding Mill starts at 1 January 2016 00:00:00, and Stocksfield starts at 1 January 2022 00:00:00. Missing timestamps or values are infrequent and are handled by forward filling on the 15 min grid before sliding-window construction. The dataset is available to download in Supplementary Materials, together with a README file describing preprocessing and reproduction steps. In this flood setup, future rainfall is treated as oracle forcing from observed records, so the experiment isolates model behaviour under controlled exogenous input rather than a fully operational weather-to-water forecasting pipeline.

### 5.1. Experimental Setup

A separate model is trained for each catchment. We compare our framework against five representative baselines. All models are optimised with Adam (initial learning rate of $3\times 10^{-4}$, $\beta_{1}=0.9$, $\beta_{2}=0.999$, $\epsilon =10^{-8}$) and early stopping based on the validation MSE. The batch size is 256, and training runs for 200 epochs with early stop. Forecast accuracy is assessed using the mean MSE, as defined in Equation (19).

Following hydrological practice, we report results on both the full test set and a high water subset, defined as samples for which their input contains at least one water level above the 95th percentile of the training distribution.

### 5.2. Results on Flood Forecasting

Table 2 compares our framework against five monolithic baselines across three catchments. Averaged across catchments, WL-MoE attains the lowest error on both the full test set and the high-water subset (MSE 0.1831 and 0.5520, respectively), corresponding to a 31.6% reduction versus the best full-set baseline (TsMixer, 0.2677) and 35.0% reduction versus the best high-water baseline (Informer, 0.8493). Per catchment, WL-MoE is best at Haltwhistle (all: 0.0785 vs. 0.0842, −6.8%; high: 0.1791 vs. 0.2120, −15.5%), Stocksfield (all: 0.4372 vs. 0.6802,−35.7%; high: 1.3952 vs. 1.9889,−29.8%) and Riding Mill (all: 0.0337 vs. 0.0350, −3.7%; high: 0.0816 vs. 0.0915, −10.8%). These patterns align with the mechanism we target: In hydrological time series, high-water episodes are far less frequent than low-flow periods, so the aggregate error is driven by the common regime; monolithic models, therefore, devote most capacity and updates to low flow and tend to underfit peaks, whereas WL-MoE routes candidate high-water windows to a specialised expert, allocating capacity and gradients to the minority regime, reducing negative transfer and variance, and thereby improving tail error—hence the larger margins on the high subset.

### 5.3. Sensitivity to Number of Experts

**Figure 4:**
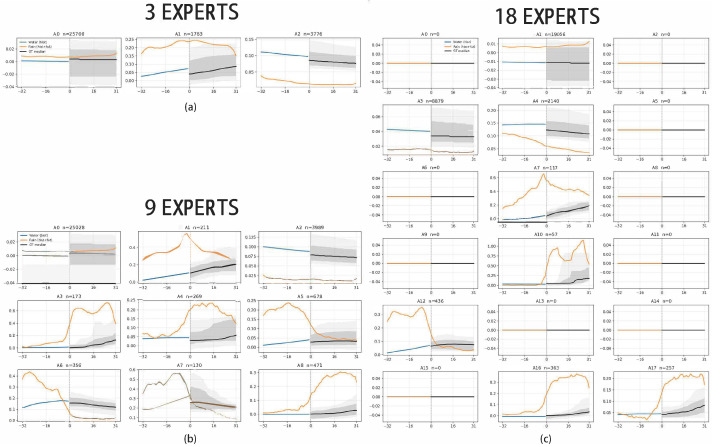
Prototype input–output profiles of WL_MoE with three, nine, and 18 experts. For three and nine experts, all experts are actively utilised, while with 18 experts, several branches remain unused, illustrating the emergence of “dead” experts. (**a**) Three experts: all branches are actively utilised, showing coarse but complete specialisation. (**b**) Nine experts: all branches remain active with clearer regime-specific profiles, indicating better-balanced specialisation. (**c**) Eighteen experts: multiple branches are inactive (e.g., panels with n=0), illustrating the emergence of dead experts under over-parameterised routing. In each panel, the black curve is the mean trajectory of routed samples, and the gray shadows indicate the band bounded by the minimum and maximum trajectories of routed samples.

Figure 4 visualises the activation patterns of WL_MoE trained with three, nine, and 18 experts. With three and nine experts, all experts are actively utilised: the gating network distributes samples across experts and each develops a meaningful specialisation (e.g., base flow, moderate flow, and high-flow regimes). In contrast, when expanded to 18 experts, a substantial fraction of experts become effectively inactive, receiving few or no assignments from the gate. These dead experts add computational overhead but contribute little to model diversity. We selected three, nine, and 18 as coarse low-/mid-/high-capacity settings to probe under-capacity, balanced capacity, and over-parameterised routing in a consistent way. This design gives a clear trend with manageable experimental cost. Intermediate values are valid alternatives: counts between 3 and 9 are expected to provide a gradual increase in specialist resolution, while counts between 9 and 18 are more likely to approach the same saturation behaviour observed at high capacity.

These results indicate that simply increasing the number of experts does not guarantee better performance. Moderate configurations (around nine experts) strike the best balance, ensuring that all experts are engaged while avoiding inefficient over-parameterisation.

### 5.4. Interpretability

In addition to forecasting accuracy, interpretability is essential for operational flood warning. We, therefore, assess WL-MoE via expert profiling; for clarity, although WL-MoE uses soft gating, we report explanations using the single expert with the highest gating probability at each step, which yields crisp insights without changing qualitative conclusions. WL-MoE experts occupy distinct hydrometeorological niches: By summarising gate outputs over the dataset, we obtain an expert-usage profile that reveals consistent specialisation rather than uniform sharing. In practice, capacity saturates at about nine experts—additional branches are rarely selected (“live” experts plateau). Given an input window, the gate produces a soft distribution over experts. We summarise explanations by pointing to the highest-probability expert, whose profile offers a simple, domain-based reason for the forecast, without probing layers. This division of labour supports physically grounded warnings rather than artefacts.

### 5.5. Case Study

To make the routing behaviour concrete, we provide two representative test windows that correspond to distinct hydrological regimes. In both cases, t=0 denotes the forecast origin: the model observes the historical segment within $X_{t}$ and predicts the subsequent *H*-step water-level trajectory. We compare a compact configuration (three experts) against a moderate configuration (nine experts), using the same input protocol and evaluation split as in Section 5.1. The goal is not to claim that a larger mixture always helps but to illustrate how additional capacity can translate into cleaner specialisation when collapse is avoided. Each panel visualises (i) the model inputs, consisting of historical rainfall and water levels up to t=0, together with the provided future rainfall over the next *H* steps and the corresponding *H*-step predicted water level (in some windows, future rainfall is zero and therefore visually unobtrusive), and (ii) for each case, the lower two panels show the profile of the expert receiving the highest probability mass.

#### 5.5.1. Case 1: Recession, No Future Rainfall

**Figure 5:**
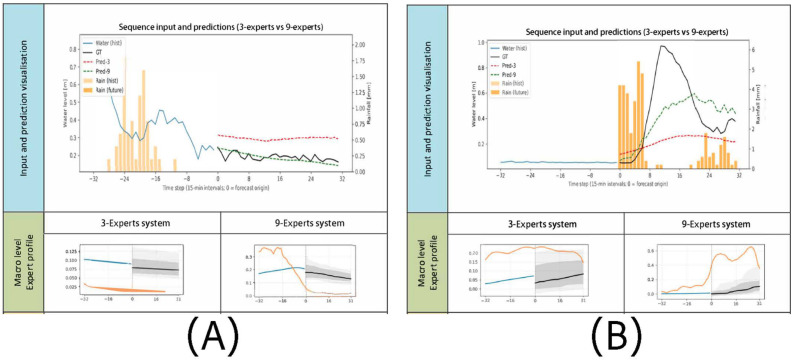
Demonstrations of two contrasting hydrological regimes. (**A**) Recession window: The top plot shows the inputs and forecasts around the forecast origin (t=0), including historical rainfall (orange bars) and water level (blue), the provided future rainfall over the next *H* steps, the ground-truth future water level (black), and the predictions from the 3-expert (red dashed) and 9-expert (green dashed) models. The 3-expert forecast drifts from the recession trajectory, whereas the 9-expert forecast better tracks the ground truth. The lower plots show the profile of the expert receiving the highest routing weight for this window in each configuration. The 3-expert system selects a broader expert whose routed windows exhibit more heterogeneous future trajectories, while the 9-expert system selects a more regime-consistent expert profile. (**B**) Rapid-onset window: Same layout as (**A**). The 9-expert model produces a faster and less attenuated rising response relative to the 3-expert model. The corresponding expert profiles indicate that the selected 9-expert prototype is more aligned with onset-like patterns, whereas the 3-expert selection reflects a broader mixture of behaviours. Together, (**A**,**B**) illustrate how WL-MoE supports regime-specific specialisation that can be inspected through expert profiling.

Figure 5A corresponds to a post-event recession. Rainfall has largely ceased around the forecast origin, and the observed water level decreases gradually over the prediction horizon, indicating a slow-release catchment response rather than a new rainfall-driven rise. In this regime, the main failure mode is overshooting or producing spurious re-acceleration due to short-term fluctuations in the recent history.

In the plot, the nine-expert system produces a smoother decay and stays closer to the ground-truth trajectory across the horizon. The associated expert profile in the lower panel shows a characteristic recession prototype, where the mean historical level exhibits a declining trend and the rainfall input remains low, and the distribution of future water levels concentrates on gradual decreases. By contrast, the three-expert system routes the window to a broader expert whose profile mixes heterogeneous behaviours, as reflected by a wider spread in the future-level band. This mismatch is consistent with the resulting prediction, which departs earlier from the observed recession shape and exhibits weaker calibration over the full horizon.

#### 5.5.2. Case 2: Rapid Onset Driven by an Abrupt Rainfall Burst

Figure 5B captures a fast-response event. Rainfall increases sharply around t=0 and continues into the forecast horizon, followed by a rapid rise in water level. Here, the forecasting difficulty is dominated by (i) reacting promptly to the onset and (ii) preserving peak amplitude without smoothing it away.

The nine-expert system responds more quickly after t=0 and produces a higher, better-timed rise relative to the three-expert system, which tends to lag and under-predict the peak. The expert profile selected by the nine-expert system aligns with an onset-type prototype: Rainfall input is elevated, and the median future water level shows a pronounced increase, with the routed future-level distribution concentrating on rising trajectories. In contrast, the expert selected by the three-expert system exhibits a less distinctive storm-response profile, with a wider and flatter future-level band, indicating that storm onsets are not cleanly separated from other regimes. This weaker specialisation is consistent with attenuation and delay in the resulting forecast under a short lead time.

## 6. Discussion

The study supports the view that weak-label-guided mixtures are most valuable as a reliability mechanism under rare, high-impact regimes rather than as a universal means of reducing the average error across all conditions. In heavily imbalanced time series, the majority of samples reflect stable dynamics, so strong aggregate metrics can coexist with systematic failures on extreme windows. The improvements observed on the high-water subset and in the flood case study therefore carry particular significance, as they are consistent with reduced regime interference and a mitigation of the underestimation behaviour that often emerges when models are trained to optimise average-case objectives.

This interpretation also reinforces a common evaluation pitfall in extreme-aware forecasting. Standard loss functions and metrics are dominated by the majority regime and implicitly favour conservative predictions that regress towards typical levels. In flood forecasting, this tendency can manifest as attenuated peaks or delayed responses, which are precisely the failure modes of greatest operational concern. Evaluating performance only on the full test distribution can therefore obscure rare but consequential errors. In this work, we partially address this issue by reporting results on both the full test set and a high-water subset that concentrates evaluation on flood-like conditions while preserving the underlying forecasting protocol.

From the perspective of optimisation dynamics, the role of weak-label guidance is to provide a stabilising scaffold that promotes early expert utilisation before the router has sufficient signal to discover a useful decomposition purely from the forecasting objective. Under severe imbalance, routed ensembles tend to concentrate probability mass on a small number of experts that fit the majority regime well. Once routing becomes highly concentrated, under-utilised experts receive limited gradient signal and fail to develop distinct prototypes, which further reinforces collapse. The proposed two-stage strategy is intended to break this feedback loop. Stage I uses coarse weak labels to encourage broader utilisation and to ensure that multiple experts are exposed to meaningful training signal. Stage II then removes the guidance so that final performance is governed by the forecasting objective, reducing the risk that artefacts of the clustering heuristic persist at inference.

The case study complements the aggregate metrics by linking individual test windows to the expert prototypes selected by the router. Instead of attributing a prediction to specific timesteps, we interpret the model through routing. For each window, we inspect which expert receives the highest routing weight and compare the window against that expert’s aggregated profile. In the recession and onset examples, the nine-expert system tends to route windows to experts whose profiles better match the pattern, whereas the three-expert system more often relies on broader experts whose routed windows cover more heterogeneous behaviours. This provides a practical audit signal. Changes in expert usage and routing concentration can be monitored to flag potential collapse or distribution shift.

## 7. Limitations

Several limitations should be acknowledged. First, the empirical setting is constrained by observability. The model inputs comprise rainfall and water level, without additional drivers such as upstream inflows, operational controls, or land-surface states. As a result, distinct physical mechanisms can induce similar input patterns, particularly during compound events. This limits identifiability and places an upper bound on performance for any purely data-driven approach operating under the same information constraints.

Second, future rainfall is treated as an available input in the forecasting protocol. In operational deployments, this quantity would typically be provided by weather forecast and therefore carries uncertainty and potential bias. Errors in rainfall forcing can propagate nonlinearly into stage predictions, especially for short-lead flood onsets where timing and intensity are sensitive. The present study should therefore be interpreted as evaluating model behaviour under given forcing, rather than fully characterising end-to-end forecast uncertainty. When oracle rainfall is replaced by operational rainfall forecasts, forecast uncertainty and bias can propagate into water-level predictions, particularly affecting short-lead peak timing and peak magnitude. Historical archived operational rainfall forecasts aligned with the study gauges and prediction horizons were not available in this study, so evaluation under realistic forecast forcing is left for future work.

Third, the weak labels used in Stage I are heuristic. They are derived from unsupervised clustering under a chosen feature mapping and are not guaranteed to correspond to physically meaningful regimes. While Stage II removes the guidance and optimises solely for the forecasting objective, the quality of the weak labels can still influence early specialisation and subsequent capacity allocation. In particular, coarse or noisy cluster structure may lead to suboptimal initial routing patterns that are only partially corrected during fine-tuning.

Fourth, the evaluation metrics used in this work do not fully align with risks. Average error measures such as the MSE or NSE do not explicitly penalise peak timing errors, peak bias, or threshold exceedance mistakes that drive false alarms and missed events. Incorporating event-based metrics and tail-weighted objectives would provide a more decision-aligned assessment, particularly for applications where the cost of underestimation is asymmetric.

Fifth, the choice of the number of experts is treated as a design parameter rather than a systematically optimised quantity. While our experiments consider several expert counts, we do not provide an exhaustive study of how expert number and capacity interact with dataset size, imbalance severity, and regime diversity. As a result, the selected configuration should be viewed as a reasonable empirical choice for the studied settings rather than a universally optimal prescription. In particular, intermediate expert counts between the reported settings (three, nine, and 18) are not exhaustively evaluated in this work.

Sixth, although experiments follow standard fixed-split benchmark protocols for comparability, this study does not include a full repeated-run statistical significance analysis across all datasets and horizons. Consequently, differences such as WL-MoE versus TsMixer MAE on Traffic should be interpreted as empirical observations under the current protocol rather than formal inferential claims. A systematic analysis with multiple independent runs, confidence intervals, and statistical tests is left for future work.

Seventh, the extreme-window partition is defined by a fixed upper-tail rule ($\tau_{0.95}$ with an exceedance-ratio cutoff of $m_{t}/H>0.05$). While this gives a clear and reproducible protocol, the current study does not quantify uncertainty of the threshold estimate or provide a full sensitivity analysis over alternative quantiles and cutoff ratios. Future work will report robustness under multiple tail-threshold settings and include uncertainty summaries for extreme-case determination.

Eighth, this paper does not provide a systematic backbone ablation across recurrent alternatives. We adopt LSTM as a fixed, classical expert architecture so that comparisons focus on the proposed MoE routing and weak-label mechanism. GRU-based experts and other backbones are valid alternatives and will be evaluated in future work.

Finally, the interpretability provided here is expert-oriented and therefore limited in scope. Routing distributions and expert profiles offer a transparent view of how the model allocates capacity across regimes and provide useful signals for monitoring and debugging. However, they do not constitute causal explanations and should not be interpreted as direct evidence of underlying physical mechanisms.

## 8. Conclusions

This paper addressed the challenge of extreme-aware time-series forecasting under severe imbalance, where models trained to minimise average error can perform well on common regimes yet fail on rare, high-impact events. We proposed WL-MoE, a weak-label-guided mixture-of-experts framework that decomposes forecasting into lightweight experts coordinated by a gating network. A two-stage training strategy was introduced to stabilise early specialisation. In Stage I, cluster-derived weak labels provide coarse guidance that encourages broader expert utilisation and mitigates routing collapse. In Stage II, guidance is removed, and the model is optimised solely for the forecasting objective, ensuring that the final predictor remains fully data-driven at inference.

Across the seven benchmark datasets at horizons H=96 and H=720, WL-MoE ranks first or second in MSE on 12/14 full-test settings and 14/14 extreme-case settings (Table 1). In the Northumberland flood study, WL-MoE is best on all three catchments for both all-window and high-water evaluation, with mean MSE reductions of 31.6% (all-window) and 35.0% (high-water) versus the strongest baselines. These results indicate that the proposed routing-and-guidance design improves tail-focused reliability while retaining competitive overall performance.

Beyond accuracy, expert-oriented profiling offers a practical transparency mechanism. By inspecting expert usage and routing concentration, practitioners can audit specialisation behaviour, monitor for collapse, and identify potential distribution shift over time.

This study also highlights that performance is ultimately bounded by data coverage and observability. With inputs limited to rainfall and water level, different physical mechanisms may be indistinguishable, and forecast quality remains sensitive to uncertainty in future rainfall forcing. Moreover, evaluation should reflect risk. Average error measures can understate peak timing and exceedance failures that matter most in high-stakes settings. These considerations motivate future work on more decision-aligned evaluation and objectives and on integrating richer drivers or uncertainty-aware forcing to further improve robustness under extremes.

## Figures and Tables

**Table 1:** Combined performance comparison across seven benchmarks. Top: Full test-set performance; bottom: extreme/high-tail subsets. Metrics are MSE/MAE at horizons H∈{96,720}. Models: WL-MoE, Informer, Autoformer, FEDformer, TimesNet, and TsMixer. Best per row is highlighted in red, and second best in blue. For cross-benchmark reporting, results are presented per dataset and per horizon, without arithmetic averaging of raw errors across datasets.

**Full Test-Set Performance**													
**Model**		**WL-MoE **		**Informer**		**Autoformer**		**FEDformer**		**TimesNet**		**TsMixer**	
**Metrics**		**MSE**	**MAE**	**MSE**	**MAE**	**MSE**	**MAE**	**MSE**	**MAE**	**MSE**	**MAE**	**MSE**	**MAE**
Weather	96	0.0035	0.0444	0.0698	0.0503	0.0066	0.0634	0.0106	0.0850	0.0108	0.0791	0.0074	0.0391
	720	0.0064	0.0636	0.0060	0.0496	0.0109	0.0853	0.0092	0.0751	0.0145	0.1154	0.0097	0.0619
Traffic	96	0.2463	0.3490	0.7738	0.6575	0.2826	0.2825	1.2357	0.8431	0.3014	0.2259	0.2641	0.2706
	720	0.2756	0.3781	1.7331	1.0657	0.2783	0.3688	1.9760	1.1740	0.3372	0.2564	0.3617	0.3665
Electricity	96	0.3603	0.4581	0.4925	0.5477	0.4009	0.4864	1.7542	1.0573	0.3982	0.5434	0.4103	0.5300
	720	0.3960	0.4682	0.8462	0.7164	0.4223	0.4980	1.3548	0.8901	0.3931	0.5487	0.3529	0.4527
ETTh1	96	0.1385	0.2480	0.1737	0.3432	0.1839	0.3362	0.1886	0.3587	0.1486	0.2371	0.1254	0.2721
	720	0.1828	0.3452	0.2065	0.3688	0.1833	0.3413	0.2233	0.3769	0.2091	0.2813	0.2292	0.2919
ETTh2	96	0.3184	0.4267	0.4150	0.5150	0.3755	0.4849	0.4064	0.5043	0.3771	0.4569	0.3533	0.4251
	720	0.4361	0.4526	0.5354	0.6035	0.5397	0.5802	0.4337	0.4575	0.4796	0.5337	0.4579	0.4844
ETTm1	96	0.0470	0.1623	0.1157	0.2584	0.1563	0.3075	0.1055	0.2580	0.0892	0.1732	0.0509	0.1637
	720	0.1740	0.3438	0.2710	0.4228	0.3810	0.4823	0.1589	0.3108	0.2922	0.3654	0.1313	0.2910
ETTm2	96	0.1343	0.2662	0.2656	0.3826	0.2973	0.4237	0.3339	0.4517	0.1774	0.3021	0.1616	0.2743
	720	0.3849	0.4971	0.6527	0.5706	0.3910	0.4879	0.4261	0.5218	0.4279	0.5399	0.4540	0.4498
**Extreme-Case Performance**													
**Model**		**WL-MoE**		**Informer**		**Autoformer**		**FEDformer**		**TimesNet**		**TsMixer**	
**Metrics**		**MSE**	**MAE**	**MSE**	**MAE**	**MSE**	**MAE**	**MSE**	**MAE**	**MSE**	**MAE**	**MSE**	**MAE**
Weather	96	0.0083	0.0755	0.1340	0.0617	0.0160	0.0626	0.0118	0.0906	0.0181	0.1349	0.0199	0.0127
	720	0.0129	0.0998	0.0153	0.0549	0.0109	0.0833	0.0137	0.1172	0.1992	0.3660	0.7345	0.6500
Traffic	96	0.4063	0.4512	0.7817	0.6617	0.4183	0.4160	1.6899	1.2849	0.5317	0.4980	0.4913	0.4828
	720	0.3648	0.4347	1.9838	1.1243	0.4101	0.3693	1.1404	1.4899	0.4569	0.4123	0.3837	0.2565
Electricity	96	0.4730	0.5274	0.5614	0.5853	0.4179	0.4239	0.9451	1.1084	0.5227	0.5861	0.5591	0.5611
	720	0.5895	0.5952	0.9426	0.7899	0.6351	0.5241	1.1086	1.0269	0.7448	0.8906	0.6699	0.6725
ETTh1	96	0.1196	0.2816	0.2979	0.4860	0.2139	0.3526	0.4844	0.6484	0.1823	0.3291	0.1245	0.2699
	720	0.1984	0.3589	0.2356	0.4102	0.2327	0.2908	0.5581	0.6082	0.3634	0.5971	0.1620	0.3334
ETTh2	96	0.3195	0.2684	0.4764	0.5827	0.4434	0.5186	0.6921	0.7169	0.3621	0.4339	0.2568	0.3846
	720	0.4588	0.4223	0.6160	0.6792	1.0149	0.8538	0.7636	0.7654	0.5165	0.6630	0.5807	0.6531
ETTm1	96	0.0468	0.1603	0.1316	0.2708	0.1613	0.3125	0.1247	0.2986	0.1221	0.2563	0.0676	0.1576
	720	0.2234	0.3986	0.2811	0.4328	0.3976	0.4947	0.1735	0.3324	0.2723	0.4665	0.2249	0.2827
ETTm2	96	0.1424	0.2490	0.3135	0.4245	0.3543	0.4368	0.3872	0.4871	0.2908	0.3532	0.1946	0.2658
	720	0.3778	0.4546	0.9045	0.7891	0.3968	0.4519	0.3917	0.4856	0.4928	0.5894	0.4280	0.5474

**Table 2:** Accuracy comparison of baseline models and WL-MoE across three catchments. Each cell displays the accuracy measurement on the full test set (All) and on the high subset, defined as samples for which their input water levels exceed the 95th percentile at any point. All values represent the average across all 32 prediction steps. Best per row is highlighted in red, and second best in blue.

	Catchment	WL-MoE All/High	Informer All/High	Autoformer All/High	FEDformer All/High	TimesNet All/High	TsMixer All/High
MSE	Haltwhistle	0.0785/0.1791	0.0843/0.2623	0.2416/0.4730	0.0875/0.3666	0.1279/0.2899	0.0842/0.2120
	Stocksfield	0.4372/1.3952	0.8979/2.1256	1.1592/1.9889	0.9587/2.1828	0.8736/2.2189	0.6802/2.2823
	Riding Mill	0.0337/0.0816	0.0475/0.1601	0.0889/0.1526	0.0350/0.1365	0.0495/0.0915	0.0387/0.1129
	Average	0.1831/0.5520	0.3432/0.8493	0.4966/0.8715	0.3604/0.8953	0.3503/0.8668	0.2677/0.8691

## Data Availability

Data are publicly available at https://environment.data.gov.uk/hydrology/explore (accessed on 1 October 2025).

## Supplementary Materials

The following supporting information can be downloaded at: https://www.mdpi.com/article/10.3390/s26051571/s1: Supplementary Data: Train and test CSV datasets for the Northumberland flood forecasting case study (Haltwhistle, Riding Mill, and Stocksfield sites), including rainfall and water level time series at 15-min resolution. README file: description of file structure, column definitions, preprocessing steps, and reproduction procedure.
